# Therapeutic advances in traditional Chinese and Tibetan medicine for high-altitude cerebral edema

**DOI:** 10.3389/fphar.2026.1796921

**Published:** 2026-05-14

**Authors:** Yuhui Gao, Yupan Luo, Liqiong Chen, Ergen Mi, Ziqi He, Bingbing Shang, Shaoguang Sui, Wanqi Zheng

**Affiliations:** 1 Department of Pediatrics, The Second Affiliated Hospital, Dalian Medical University, Dalian, China; 2 Laboratory Research and Teaching Department of Comparative Medicine, Dalian Medical University, Dalian, China; 3 Research and Teaching Department of Comparative Medicine, Dalian Medical University, Dalian, Liaoning, China; 4 Emergency Department, The Second Affiliated Hospital, Dalian Medical University, Dalian, China

**Keywords:** blood–brain barrier damage, botanical drug, high-altitude cerebral edema, hypoxia, Tibetan medicine, traditional Chinese medicine

## Abstract

**Introduction:**

High-altitude cerebral edema (HACE) is an exacerbation of acute mountain sickness, but also a distinct disease characterized by vasogenic and cytotoxic brain edema. Exploring alternative therapies, such as traditional Chinese medicine (TCM) and Tibetan medicine (TM), is important. We aimed to provide a comprehensive overview of the existing research on the therapeutic effects and underlying molecular mechanisms of TCM and TM, encompassing herbal formulations, single botanical drugs, and their bioactive *metabolites*.

**Methods:**

We employed keywords such as “TCM,” “TM,” “Botanical drug,” “HACE,” and “Hypoxia” to systematically retrieve studies on the therapeutic use of TCM and TM for HACE published up to May 2025, in databases including Web of Science, PubMed, China National Knowledge Infrastructure (CNKI) and Wanfang Data.

**Results:**

We conducted analysis of 97 research articles, encompassing the latest advancements in the treatment of HACE using 4 TCM formulations, 12 single botanical drugs of TCM, 3 TM formulations, and five single botanical drugs of TM. Sources, effects, and molecular mechanisms of the TCM and TM were summarized.

**Discussions:**

We report the progress of traditional botanical drugs and their formulations derived from TCM and TM in the management of HACE, based on the medical practices and materia medica from the high mountain regions in Eastern Asia. We provide a foundation for the clinical application and further development of these medicinal resources.

## 1 Introduction

High-altitude cerebral edema (HACE) is a severe manifestation of acute mountain sickness (AMS), which typically affects those who ascend too quickly to high-altitude regions. HACE is characterized by sudden onset, rapid worsening of symptoms, and a notably elevated mortality risk (Hackett and Roach, 2004). The incidence of HACE is increasing with the increase in high-altitude tourism, work, and military activities, particularly in high-altitude regions, and poses a severe threat to human health (Hackett and Roach, 2001; West, 2012; Luks and Hackett, 2022). Empirical evidence suggests that HACE primarily affects individuals who rapidly ascend to >3,000 m above sea level, with a notable and significant surge in incidence rate observed when altitudes surpass the 5,000 m threshold (Basnyat and Murdoch, 2003). The pathogenesis of HACE has not yet been fully elucidated; however, it is mainly associated with hemodynamic changes in brain tissue under hypoxic conditions, increased blood–brain barrier (BBB) permeability, oxidative stress responses, and inflammatory factor overexpression (Bailey et al., 2004; Luks, 2015). Additionally, individual susceptibility, genetic factors, and adaptability to high-altitude environments play important roles in the pathogenesis of HACE (Julian, 2017). Current allopathic medical treatments for HACE mainly involve oxygen therapy and pharmacological reduction of intracranial pressure, and have certain limitations (Bartsch et al., 2004; Basnyat and Murdoch, 2003). Although oxygen therapy can alleviate hypoxia, high-flow oxygen administration can induce oxygen toxicity and lung injury. Moreover, the limited availability of oxygen supply equipment in high-altitude areas makes maintaining stable therapeutic effects difficult. Pharmacological reduction of intracranial pressure (e.g., with mannitol and furosemide) may cause electrolyte disturbances, renal dysfunction, and hypercoagulability, and repeated use can lead to tolerance, thereby failing to address the root cause of the disease (Wei et al., 2019).

China has the world’s largest expanse of high-altitude territories. Regions situated at altitudes exceeding 2,500 m constitute more than a quarter of the country’s total land area. In stark contrast to the plains, high-altitude zones are marked by distinctive environmental features, including low atmospheric pressure, reduced oxygen partial pressure, frigid temperatures, intense ultraviolet (UV) radiation, and an arid climate. Collectively, these conditions exert a profound influence on human adaptability and overall health (Huerta-Sánchez et al., 2014). Traditional Chinese Medicine (TCM) offers a distinctive holistic paradigm for understanding health and disease. According to TCM principles, health is maintained through the unobstructed circulation of *Qi* (vital energy) and fluids within the human body, coupled with the delicate equilibrium between Yin and Yang energies—a dynamic interplay that underpins physiological harmony (Tang et al., 2008; Wang and Xiong, 2013).

Tibetan Medicine (TM), a medical tradition that evolved within the harsh and hyperbaric ecological niche of the Qinghai–Tibetan Plateau, has cultivated distinctive theoretical frameworks and therapeutic protocols for high-altitude-related pathologies, including HACE. Based on millennia of adaptations to the plateau’s extreme conditions—characterized by hypobaric hypoxia (HH), overload of UV radiation, and drastic diurnal temperature fluctuations—this indigenous medical system articulates unique pathophysiological interpretations and interventional strategies that diverge from, yet complement, contemporary biomedical approaches (Zhang, 2007; Fu et al., 2020). TM, similar to TCM, is a holistic system that emphasizes the interconnection between the body, mind, and environment, and aims to restore balance and harmony to maintain health and treat disease.

Contemporary scientific investigations have validated the fact that traditional botanical drugs and their formulations derived from TCM and TM possess distinctive benefits for preventing and treating HACE by modulating mechanisms such as anti-hypoxia, hypoxic pulmonary hypertension reduction, angiogenesis and vasoconstriction inhibition, decreased blood-flow resistance, reduced cell permeability, inflammatory response alleviation, and anti-oxidative stress (Feng et al., 2013). For instance, TCM metabolites, such as tanshinone-IIA and ginsenoside Rg1, have been scientifically verified to effectively regulate antioxidant enzymatic activity and inhibit the expression of proinflammatory factors, thereby significantly reducing the brain damage caused by high-altitude hypoxia (Jiang et al., 2022; Xie et al., 2022). TM, with its unique theoretical system and rich pharmacological resources, has also accumulated valuable experience in the prevention and treatment of high-altitude diseases. For example, the Duoxuekang capsule can regulate the mitogen-activated protein kinase (MAPK) signaling pathway, reduce whole-blood viscosity, decrease oxidative damage, and improve brain injury-related complications of high-altitude polycythemia (Basnyat and Murdoch, 2003). Furthermore, the metabolites of TM, such as *Rhodiola* and *Saussurea involucrata*, have demonstrated good anti-hypoxia and neuroprotective effects (Ma et al., 2011; Wang et al., 2019).

Despite the important role of modern medicine in the emergent treatment of HACE, TCM and TM offer new ideas and methods for the prevention and treatment of this condition, with their multi-targeted and holistic regulatory advantages. However, currently, the clinical evidence supporting traditional drug treatment for HACE is still in the early stages overall, and the number of high-quality large-sample randomized controlled trials (RCTs) is limited (Huo et al., 2021).

**FIGURE 1:**
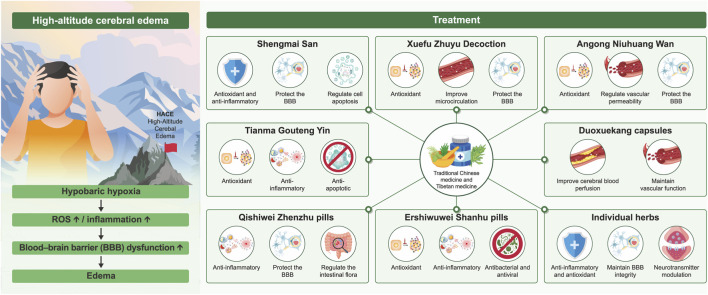
Summarizing the core therapeutic effects of TCM and TM formulations on HACE.

In this review, we assessed the latest research advances in the application of TCM and TM for the treatment of HACE, with the aim of providing evidence-based references for future scientific research and clinical practice. A comprehensive literature search was performed across multiple academic databases. Specifically, the English search terms including “Traditional Chinese Medicine”, (Tang et al., 2008), “Tibetan Medicine”, “*Botanical drug*”, “HACE”, and “Hypoxia” were employed in Web of Science and PubMed. Meanwhile, conceptually consistent Chinese search terms were utilized in the China National Knowledge Infrastructure (CNKI) and the Wanfang Data to ensure the comprehensiveness of literature retrieval. The literature screening process was conducted in two sequential stages. Initially, a preliminary screening was carried out based on titles and abstracts to exclude studies with no obvious relevance to the research topic. Subsequently, the full texts of the preliminarily screened literature were obtained and meticulously reviewed. The inclusion criteria were defined as follows: studies with a clear focus on HACE and its key pathophysiological pathways; interventions involving extracts, active metabolites, or compound formulations of TCM or TM; and provision of specific pharmacological mechanism data or clinical efficacy outcomes. Conversely, the exclusion criteria encompassed review articles, conference abstracts, and studies with incomplete or unavailable data. In this search, we covered all relevant articles published up to May 2025 regarding the use of TCM and TM in HACE treatment. Finally, we systematically compiled and summarized the eligible studies to present a holistic overview of the current landscape of TCM and TM applications in HACE management (Tables 1, 2) (Figure 1).

**TABLE 1:** Mechanism of action of individual treatments in traditional Chinese medicine and Tibetan medicine for HACE.

Botanical drug	Active metabolite	Signaling pathway
*Eleutherococcus senticosus*	Eleutheroside B	JAK2/STAT3
	Eleutheroside E	
*Ligusticum chuanxiong*	Tetramethylpyrazine	NRF2
	Ligustrazine hydrochloride	
*Astragalus membranaceus*	Astragaloside IV	Nrf2/HO-1
		mTOR
		Nrf2
*Panax notoginseng*	Notoginsenoside	Wnt/β-catenin
		HIF1-α/VEGFA/VEGFR2 axis
		JNK/ERK/P38
		PI3K/AKT/mTOR
*Salvia miltiorrhiza*	Tanshinone IIA	Nrf2
*Coptis chinensis*	Berberine	NF-κB
		AMPK/PGC1α
		AMPK
*Pheretima aspergillum*	—	Nuclear factor-κB p65
*Gastrodia elata Bl.*	Gastrodin	NRF2/HO-1
*Ganoderma lucidum*	Ganoderma polysaccharides	ERK1/ERK2
*Curcuma longa L.*	Curcumin	NF-κB/VEGF/MMP-9
	Tetrahydrocurcumin	
*Gymnadenia conopsea (L.) R. Br.*	—	HIF-1
		PI3K-Akt
		NF-kappa B
*Gynostemma pentaphyllum*	Gypenoside	NF-κB
*Potentilla anserina L.*	Potentilla anserina	NF-κB
	Polysaccharide	HIF-1α
	Euscaphic acid	ERK1/2
	Tormentic acid	PI3K/AKT
*Rhodiola crenulata*	Salidroside	NF-κB/NLRP3
		AMPK/Sirt1
		HIF-1α/microRNA 210/ISCU1/2 (COX10)
*Crocus sativus*	Crocin	—
*Saussurea involucrata*	—	—
*Brassica rapa L.*	Brassica rapa L. polysaccharides	PI3K/Akt/HIF-1α
		PI3K/Akt/mTOR

**TABLE 2:** Some effects of prescriptions of TCM and TM in the treatment of HACE.

Prescription	Source species/Botanical drugs	Function
Shengmai San	Radix ginseng (*Panax ginseng* C.A.Mey.) | Araliaceae	Antioxidant
	Radix Ophiopogonis (*Ophiopogon japonicus* (Thunb.) Ker Gawl.) | Asparagaceae	Anti-inflammatory
		Protect the blood–brain barrier
		Trophic nerve
	Fructus Schisandrae (*Schisandra chinensis* (Turcz.) Baill.) | Schisandraceae	Regulate cell apoptosis
Xuefu Zhuyu decoction	Semen persicae (*Prunus persica* (L.) Batsch) | Rosaceae	Antioxidant
	Radix angelicae sinensis (*Angelica sinensis* (Oliv.) Diels) | Apiaceae	Improve microcirculation
	Radix paeoniae rubra (*Paeonia lactiflora Pall.*) | Paeoniaceae	Protect the blood–brain barrier
	Radix Achyranthis Bidentatae (*Achyranthes bidentata* Blume) | Amaranthaceae	Platelet activation
	Flos carthami (*Carthamus tinctorius* L.) | Asteraceae	​
	Rhizoma chuanxiong (Ligusticum chuanxiong Hort.) | Apiaceae	​
	Radix bupleuri (*Bupleurum chinense* DC.) | Apiaceae	​
	Fructus Aurantii (*Citrus aurantium* L.) | Rutaceae	​
	Radix Rehmanniae (*Rehmannia glutinosa* (Gaertn.) Libosch. ex DC.) | Orobanchaceae	​
	Radix Platycodonis (*Platycodon grandiflorus* (Jacq.) A.DC.) | Campanulaceae	​
	Radix Glycyrrhizae (*Glycyrrhiza glabra* L.) | Fabaceae	​
Angong Niuhuang wan	Bovis Calculus (*Bos taurus domesticus* Gmelin) | Bovidae	Antioxidant
	Cornu Bubali (*Bubalus bubalis* Linnaeus) | Bovidae	Regulate vascular permeability
	Moschus (*Moschus berezovskii* Flerov) | Moschidae	Protect the blood–brain barrier
	Margarita (*Hyriopsis cumingii* lea) | Unionidae	​
	Cinnabaris | *Mineral*	​
	Realgar | *Mineral*	​
	Rhizoma coptidis (*Coptis chinensis* Franch.) | Ranunculaceae	​
	Radix scutellariae (*Scutellaria baicalensis* Georgi) | Lamiaceae	​
	Fructus gardeniae (*Gardenia jasminoides* J.Ellis) | Rubiaceae	​
	Radix Curcumae (*Curcuma longa* L.) | Zingiberaceae	​
	Borneolum (*Dryobalanops aromatica* C.F.Gaertn.) | Dipterocarpaceae	​
Tianma Gouteng yin	Radix Gastrodiae (*Gastrodia elata* Blume) | Orchidaceae	Antioxidant
	Uncariae ramulus cum uncis (*Uncaria rhynchophylla* (Miq.) Miq.) | Rubiaceae	Anti-inflammatory
	Concha Haliotidis | Haliotidae	Trophic nerve
	Fructus gardeniae (*Gardenia jasminoides* J.Ellis) | Rubiaceae	Anti-apoptotic
	Radix scutellariae (*Scutellaria baicalensis* Georgi) | Lamiaceae	​
	Radix Achyranthis Bidentatae (*Achyranthes bidentata* Blume) | Amaranthaceae	​
	Cortex eucommiae (*Eucommia ulmoides* Oliv.) | eucommiaceae	​
	Herba Leonuri (*Leonurus japonicus* Houtt.) | Lamiaceae	​
	Taxilli herba (*Taxillus chinensis* (DC.) Danser) | Loranthaceae	​
	Caulis Polygoni Multiflori (*Pleuropterus multiflorus* (Thunb.) Turcz. ex Nakai) | Polygonaceae	​
	Poria cum radix Pini (*Poria cocos* (Schw.) Wolf) | Polyporaceae	​
Duoxuekang capsules	Radix Rhodiolae (*Rhodiola crenulata* (Hook.f. and Thomson) H.Ohba) | Crassulaceae	Improve cerebral blood perfusion
	Fructus Phyllanthi (*Phyllanthus emblica* L.) | Phyllanthaceae	Inhibit oxidative stress injury
	Fructus Hippophaes (*Hippophae rhamnoides* L.) | elaeagnaceae	Maintain the integrity of cerebrovascular endothelial cells
	Rhizoma Zingiberis (*Zingiber officinale* Roscoe) | Zingiberaceae	Maintain vascular function
Qishiwei Zhenzhu pills	Margarita (*Hyriopsis cumingii* lea) | Unionidae	Anti-inflammatory
	Lignum Santali Albi (*Santalum album* L.) | Santalaceae	Protect the blood–brain barrier
	Lignum Dalbergiae Odoriferae (*Dalbergia odorifera* T.C.Chen) | Fabaceae	Regulate the intestinal flora
	Dzi Bead | Mineral	Trophic nerve
	Stigma croci (*Crocus sativus* L.) | Iridaceae	​
	Bovis Calculus (*Bos taurus domesticus* Gmelin) | Bovidae	​
	Moschus (*Moschus berezovskii* Flerov) | Moschidae	​
Ershiwuwei Shanhu pills	Corallium | Coralliidae	Antioxidant
	Margarita (*Hyriopsis cumingii* lea) | Unionidae	Anti-inflammatory
	Lapis Lazuli | *Mineral*	Analgesic
	Margaritifera concha | Margaritiferidae	Antibacterial
	Fructus Terminaliae Chebulae (*Terminalia chebula* Retz.) | Combretaceae	Antiviral
	Radix Aucklandiae (Dolomiaea costus (Falc.) Kasana and A.K.Pandey) | Asteraceae	Immunosuppressive
	Flos carthami (*Carthamus tinctorius* L.) | Asteraceae	​
	Flos Caryophylli (*Syzygium aromaticum* (L.) Merr. and L.M.Perry) | Myrtaceae	​
	Lignum Aquilariae (*Aquilaria malaccensis* Lam.) | Thymelaeaceae	​
	Cinnabaris | *Mineral*	​
	Os Draconis | Fossilia Ossis Mastodi	​
	Calamina | *Mineral*	​
	Nao Shi *| Mineral*	​
	Magnetitum | Mineral	​
	Hematitum | Mineral	​
	Semen Sesami (Sesamum indicum L.) | Pedaliaceae	​

## 2 Pathological mechanisms of HACE

The core damage associated with HACE stems from hypoxia, which is directly caused by the diminished atmospheric partial pressure of oxygen at elevated altitudes. Decreased oxygen availability triggers a series of physiological responses aimed at maintaining cerebral oxygen delivery; however, paradoxically, these compensatory mechanisms may contribute to HACE pathogenesis (Lawley et al., 2016). The initial response to hypoxia includes increased cerebral blood flow to compensate for reduced blood oxygen content in the blood. In the early stages of high-altitude hypoxic exposure, cerebral oxygen delivery is initially maintained through increased velocity and diameter of the middle cerebral artery, as ascertained from magnetic resonance imaging findings (Sagoo et al., 2017). Although vasodilation initially promotes oxygen supply, it may also lead to increased cerebral blood volume, which in turn may elevate intracranial pressure (ICP). Despite ongoing research and controversy regarding the exact role of ICP in HACE and related altitude illnesses, a general consensus that dysregulation or pathological fluctuations in ICP dynamics play a significant role in symptom manifestation exists (Wilson et al., 2014). Studies have reported that ICP normalizes over time during altitude exposure; however, the interplay between ICP fluctuations, vascular reactivity, and cerebrospinal fluid dynamics appears to be central to the development of altitude-related neurological syndromes (Hackett, 1999). Furthermore, hypoxia increases BBB permeability, which is primarily manifested by reduced expression of tight-junction proteins, and results in impaired BBB function, and, ultimately, cerebral edema (Turner et al., 2021). Elevated levels of hypoxia-inducible factor-1α (HIF-1α) induce increased expression of the vascular endothelial growth factor (VEGF) which enhances vascular permeability and exacerbates cerebral edema. Hypoxia affects cerebrospinal fluid circulation and clearance, resulting in cerebrospinal fluid retention, induces the release of proinflammatory cytokines (e.g., TNF-α, IL-1β, IL-6), activates microglia, intensifies brain tissue damage, and increases the generation of reactive oxygen species (ROS) which damage neurons and the vascular endothelium (Chen et al., 2024).

## 3 Progress in research on TCM-based treatment of HACE

### 3.1 Understanding of HACE in TCM

TCM is guided by a holistic view that considers human health dependent on the dynamic coordination between the internal and external environments. Its core theory focuses on the balance of the body’s functional state and emphasizes the enhancement of self-regulatory capabilities, rather than mere symptom management, to achieve disease prevention and treatment. In the TCM system, HACE is considered an acute condition that is triggered by an imbalance between the body’s adaptability and a unique natural environment. The TCM views the inhospitable high-altitude environment, marked by low atmospheric pressure, oxygen scarcity, and coldness, as “environmental factors that can cause diseases.” When the human body is exposed to such an environment and its self-regulatory capacity proves insufficient (e.g., weak cardiopulmonary function or low metabolic efficiency), oxygen delivery is obstructed, and energy metabolism becomes abnormal. TCM has observed that this imbalance frequently manifests as symptoms, such as headache, altered consciousness, and vomiting, which are closely related to cerebral hypoxia and fluid metabolic disorders, indicating a pathological correspondence that has been confirmed to be associated with brain tissue edema and microcirculation disorders in modern medical research.

TCM treatment emphasizes the combination of “urgent symptom relief” and “long-term adaptation enhancement.” TCM focuses on strengthening cardiopulmonary functional reserves and increasing the body’s tolerance threshold to hypoxic environments by using medicinal plants such as Rhodiola and *Salvia miltiorrhiza,* to improve oxygen utilization efficiency and promote cerebral blood circulation, combining aromatic substances to regulate nervous system function for rapid relief of headache and coma. This treatment strategy, which targets acute symptoms and reinforces physiological foundations, reflects the core concepts of “systemic regulation” and “preventive intervention” of TCM and offers a unique multidimensional solution for HACE.

### 3.2 Individual Chinese botanical drugs

*Eleutherococcus senticosus*, a classic tonic botanical drug belonging to the Araliaceae family, has a medicinal legacy that traces back to the ancient text “Shennong Bencao Jing.” Over time, it has served as a functional food ingredient and a key component in numerous TCM remedies. Its traditional uses include invigorating the liver and kidneys, restoring vitality, fortifying bones, boosting appetite, and improving memory. It also demonstrates beneficial pharmacological impacts on the cardiovascular, central nervous, and immune systems (Jia et al., 2021). Modern pharmacological studies have reported that the roots, stems, and rhizomes of *Eleutherococcus senticosus* are rich in active metabolites, such as eleutheroside B, eleutheroside E, and isofraxidin, which regulate multiple targets that are involved in antioxidant damage, anti-inflammatory effects, neurotransmitter modulation, improvement of neuronal growth, and anti-apoptosis, thereby demonstrating unique potential in the prevention and treatment of neurological diseases and high-altitude illnesses (Li X. T. et al, 2022). *In vivo* experimental research has revealed that eleutheroside B can significantly inhibit the abnormal activation of the JAK2/STAT3 signaling pathway in a rat model of HACE, and thereby reduce oxidative stress damage and suppress the neuroinflammatory cascade; this indicates its intervention value in high-altitude hypoxia-related pathological mechanisms (He et al., 2024).

*Ligusticum chuanxiong* is a perennial botanical drug of the Apiaceae family and its dried underground stem (rhizome) is employed in medicinal applications. This botanical drug is renowned for its abilities to enhance blood circulation, regulate the flow of qi (vital energy in TCM), expel wind (a pathogenic factor in TCM), and alleviate pain. Clinically, it has been used extensively in the prevention and management of cardiovascular and cerebrovascular ailments, and blood stasis-related conditions. Modern pharmacological studies have reported that tetramethylpyrazine (TMP), an active metabolite in *Ligusticum chuanxiong*, possesses various physiological functions, including antioxidant capabilities, anti-inflammatory actions, anti-apoptotic characteristics, the ability to regulate autophagy, vasodilatory functions, modulation of angiogenesis, inhibition of mitochondrial damage, safeguarding of endothelial cells, and mitigation of vascular smooth muscle cell proliferation and migration, and also offers neuroprotective benefits (Lin et al., 2022). Further mechanistic studies have reported that in *in vivo* experiments using a mouse model of focal ischemic stroke (IS), TMP upregulates the expression of monocyte chemoattractant protein-induced protein one to inhibit neuroinflammatory responses, while protecting the integrity of the BBB (Jin et al., 2021). Ligustrazine hydrochloride (LH), another important metabolite of *Ligusticum chuanxiong*. In *in vivo* experiments, it can trigger the activation of the nuclear factor E2-related factor 2 (NRF2) signaling cascade to inhibit ferroptosis and resist oxidative stress, exerting neuroprotective effects on rats with HACE (Han et al., 2025). These regulatory mechanisms that act on multiple targets offer a scientific underpinning for the clinical utilization of *Ligusticum chuanxiong* for treating HACE.

The dried root of *Astragalus membranaceus*, a leguminous plant with medicinal and edible properties, is used in medicine and widely applied clinically for the treatment of cardiovascular and cerebrovascular diseases. Astragaloside IV (AS-IV), an active metabolite extracted from *Astragalus membranaceus*, has therapeutic effects on central nervous system (CNS) diseases. In an *in vivo* experiment using a brief middle cerebral artery occlusion (MCAO) model in rats, AS-IV can regulate nuclear inflammation and ferroptosis through the Nrf2/HO-1 signaling pathway, thereby improving brain edema and reducing neuronal death (Zhang et al., 2023). When rats are exposed to high-altitude hypoxia, their cognitive abilities can be significantly impaired. In an *in vivo* experimental study, rats were exposed to a simulated high-altitude HH chamber. The aqueous extract of *Astragalus membranaceus* can significantly improve the cognitive function of hypoxic rats, with potential mechanisms related to the alleviation of oxidative stress, reduction of free radicals and metabolite accumulation, and activation of the mechanistic target of rapamycin (mTOR) signaling pathway (Du et al., 2022). Furthermore, A rat *in vivo* experiment using cerebral ischemia/reperfusion (I/R) injury as a model reported that AS-IV isolated from *Astragalus membranaceus* can prevent brain edema and BBB breakdown, through the upregulation of matrix metalloproteinase-9 (MMP-9) and aquaporin 4 (AQP4), mediated via the activation of the Nrf2 signaling pathway (Li M. et al, 2013; Li et al., 2018). The aforementioned mechanisms provide a pharmacological basis for the preventive and therapeutic effects of *Astragalus membranaceus* on brain injuries associated with high-altitude hypoxia, and suggest its potential value as a neuroprotective agent.

*Panax notoginseng*, a medicinal plant of the Araliaceae family, was first recorded in the Ming Dynasty’s “Compendium of Materia Medica.” Its dried root and rhizome have hemostatic, anti-thrombotic, anti-inflammatory, and analgesic effects, and are widely used clinically for the prevention and treatment of cardiovascular, cerebrovascular, and hemorrhagic diseases. Panax Notoginseng Saponins (PNS), the core active metabolite of *Panax notoginseng*, can regulate angiogenesis and endothelial function through multi-target mechanisms, and demonstrates potential applicability in the treatment of HACE. The *in vitro* cell-based mechanistic studies have reported that PNS can upregulate the angiogenic capacity of endothelial progenitor cells by activating the Wnt/β-catenin signaling pathway and synergistically regulate the HIF1-α/VEGFA/VEGFR2 signaling axis to promote angiogenesis, thereby improving cerebral microcirculation disorders (Zhu et al., 2021; Xiao et al., 2024). Additionally, another *in vitro* cell-based experiment showed that PNS can inhibit excessive activation of the JNK/ERK/P38 pathway in the oxygen–glucose deprivation/reoxygenation (OGD/R) model, while enhancing the cell-survival effects mediated by the PI3K/AKT/mTOR signaling pathway. This significantly reduces oxidative damage and apoptosis of brain microvascular pericytes, and increases the release of pericyte proangiogenic regulators (Zhang T. et al, 2022). These mechanisms provide molecular pharmacological evidence for the application of *Panax notoginseng* in the repair of high-altitude hypoxia-related cerebral microcirculation disorders and neuroprotection.

*Salvia miltiorrhiza*, a perennial herb of the Lamiaceae family, is extensively employed in clinical practice for the management and treatment of cardiovascular and cerebrovascular disorders. Contemporary pharmacological research has demonstrated that the lipophilic active metabolites of *Salvia miltiorrhiza*, the tanshinone compounds, possess multifaceted neuroprotective effects, such as anti-inflammatory, antioxidant, anti-apoptotic, and BBB protective properties, which can alleviate the pathological progression of HACE (Subedi and Gaire, 2021). Tanshinone IIA (Tan-IIA) has attracted attention for its unique ability to cross the BBB, and its multi-target neuroprotective activities (Sherawat and Mehan, 2023). A study including both *in vivo* and *in vitro* experiments has reported that Tan-IIA can prevent lipopolysaccharide (LPS)-induced brain damage by activating the Nrf2 signaling pathway and rescuing tight-junction proteins in microvascular endothelial cells, thereby inhibiting oxidative stress and inflammatory responses to protect the BBB (Wang et al., 2022). An *in vitro* cell-based experiment using the human BBB model has reported that Tan-IIA can safeguard the BBB against hypoxia–reoxygenation injury, which is linked to leukocytes, by reducing leukocyte activation and curbing the harmful effects of leukocyte-generated substances (Zhang W. J. et al, 2010). Research based on network pharmacology indicates that Tan-IIA can effectively suppress apoptosis and boost the expression levels of VEGF-A, P-MEK1/2, and P-ERK1/2 proteins. Through these actions, it safeguards endothelial cells, stimulates angiogenesis, and enhances microcirculation, ultimately alleviating brain edema (Li Z. K. et al, 2022). The above evidence suggests that tanshinone components, especially Tan-IIA, have significant potential in the repair of cerebral microcirculation disorders and neurofunctional restoration in hypoxic environments at high altitudes.

*Coptis chinensis* is a perennial herb of the Ranunculaceae family and its dried rhizome is used medicinally for its anti-inflammatory, antibacterial, and detoxifying activities. *Coptis chinensis* is used clinically to treat inflammatory diseases of the digestive system, purulent skin diseases, and systemic inflammatory response syndromes. Modern pharmacological studies have reported that berberine, the core active metabolite of *Coptis chinensis*, exhibits antioxidant stress, anti-neuroinflammatory, and dual regulatory effects on cell apoptosis and autophagy, and promotes angiogenesis through the regulation of multiple signaling pathways, thereby showing unique advantages in the treatment of HACE (Sunhe et al., 2024). *In vivo* experimental research on mice findings reveal that berberine can inhibit the activation of the NF-κB signaling cascade and curb the expression of proinflammatory agents, such as TNF-α and IL-6, helping to mitigate neuroinflammatory damage (Liu et al., 2017). Additionally, The results of the *in vivo* experimental study based on the mouse model of cerebral hemorrhage revealed that the neuroprotective effects of berberine on neuroinjury and BBB damage may be mediated through the inhibition of inflammation and activation of the AMPK/PGC1α signaling pathway (Wu et al., 2022). Berberine can activate the AMPK pathway and thereby reduce the production of mitochondrial ROS and improve energy metabolism. Furthermore, it has the capacity to upregulate the activity of pivotal antioxidant enzymes, including superoxide dismutase (SOD), catalase, and glutathione peroxidase, which in turn protects cells from oxidative harm (García-Muñoz et al., 2024). The abovementioned molecular mechanisms suggest that *Coptis chinensis* and its active metabolite berberine, through their dual effects of antioxidant stress and anti-neuroinflammation, constitute a potential intervention strategy for cerebral microcirculation disorders and BBB protection in hypoxic environments at high altitudes.

*Pheretima aspergillum*, the dried body of the earthworm, first recorded in the “Shennong Bencao Jing,” has anticonvulsant, neuroprotective, bronchodilatory, anti-inflammatory, and diuretic functions. It has prominent therapeutic effects on various inflammatory diseases such as asthma, cough, and fever. Modern studies have reported that the protein-free Guang-Pheretima decoction (PF-GPD) can inhibit the release of inflammatory factors IL-1β and IL-6, regulate the homeostasis of microcirculation, and play an important role in the intervention of HACE pathology (Huang et al., 2018). Additionally, Shuxuetong injection, composed of Pheretima extract, protects brain microvascular endothelial cells in the OGD/R model by reducing mitochondrial superoxide production, inhibiting inflammatory responses, and suppressing the vascular endothelial growth factor, extracellular signal-regulated kinase 1/2, and nuclear factor-κB p65 signaling pathways (Sun et al., 2019). The aforementioned mechanisms suggest that *Pheretima aspergillum* and its compound formulations can modulate neuroinflammation and microcirculatory dysfunction, offering a potential therapeutic strategy for mitigating oxidative damage in brain tissue, and restoring the BBB function under high-altitude hypoxic conditions.

*Gastrodia elata Bl.*, a perennial herb of the Orchidaceae family, has anticonvulsant, neuroprotective, analgesic, and anti-neuroinflammatory effects, and is used clinically to intervene in neurological diseases such as vertigo, headache, and convulsions. Gastrodin is the core active metabolite that following intravenous administration to rats, could rapidly enter the brain; moreover, the main metabolite of gastrodin (p-hydroxybenzyl alcohol) has very low concentrations in cerebrospinal fluid and plasma (Wang et al., 2008). An experimental study based on an IS mouse model and an OGD/R cell model has reported that neutral polysaccharides derived from *Gastrodia elata* can inhibit ferroptosis-mediated neuroinflammatory responses by activating the NRF2/HO-1 signaling pathway, significantly reducing the apoptosis rate of HT22 cells in the OGD/R model, and alleviating neuronal damage related to brain edema (Zhang et al., 2024). The aforementioned research indicates that *Gastrodia elata Bl*. and its bioactive *metabolites* can regulate neuroinflammatory responses, oxidative stress, and cellular death pathways, demonstrating remarkable efficacy in the treatment of HACE.

*Ganoderma lucidum* is a fungus of the Ganodermataceae family and its fruiting body is used medicinally for its immunomodulatory, neuroprotective, antioxidant, anti-inflammatory, anticancer, and antibacterial effects. Modern studies have reported that Ganoderma polysaccharides, as its core active metabolites, can exert antioxidant and neuroprotective effects by inhibiting mitochondrial oxidative stress (Seweryn et al., 2021). An *in vivo* experimental study based on rat HH test model has reported that the aqueous extract of *Ganoderma lucidum* can improve neurotransmitter levels in HH animal models, prevent the surge of glucocorticoids and α-synuclein, induce the upregulation of CREB/p-CREB/BDNF expression through ERK1/ERK2 to improve synaptic plasticity, and maintain redox homeostasis, to improve HH-related spatial memory deficits and neuronal damage (Sharma and Tulsawani, 2020). The abovementioned mechanisms suggest that *Ganoderma lucidum* and its active metabolites, through multi-dimensional regulation of neurotransmitter networks, oxidative stress responses, and synaptic remodeling, may provide a potential intervention strategy for HACE-induced cognitive dysfunction and brain tissue damage.

*Curcuma longa L.—*a perennial herb of the Zingiberaceae family, having anti-inflammatory, antioxidant, antitumor, vasoactive, and analgesic effects—is used clinically to treat microcirculation disorders, primary dysmenorrhea, and osteoarthritis, and as an adjuvant treatment for malignant tumors. Curcumin is a member of the curcuminoid family and is the main active metabolite in *Curcuma longa*, which can improve BBB damage mediated by ONOO(-) (Jiang et al., 2007). Modern pharmacological studies have reported that tetrahydrocurcumin, the main active metabolite of *Curcuma longa*, has significant potential in the pathological intervention of HACE. An *in vivo* experiment based on the acute HH mouse model has confirmed that prophylactic administration of tetrahydrocurcumin can alleviate acute HH-induced brain edema, reduce the levels of inflammatory factors IL-1β and TNF-α, increase SOD activity to enhance hypoxia resistance, and inhibit the NF-κB/VEGF/MMP-9 pathway to reduce HH-induced brain edema and inflammation, thereby exerting neuroprotective effects (Pan et al., 2020). The above-cited studies report that *Curcuma longa* and its active metabolites, through multi-target regulation of inflammatory responses and BBB protection, may provide a novel strategy for the pathological intervention of brain edema in hypoxic environments at high altitudes.

*Gymnadenia conopsea* (L.) R. Br., a perennial terrestrial herb of the Orchidaceae family, has nourishing, antioxidant, antiviral, immunomodulatory, anti-allergic, anti-gastric ulcer, sedative, and hypnotic effects, and is traditionally used to replenish and regulate qi and blood and relieve cough and asthma, with botanical drugs sourced from the Tibetan Plateau being the most effective. Modern studies have reported that *Gymnadenia conopsea* has potential interventional value for brain damage induced by high-altitude hypoxia (Shang et al., 2017). The results of an experiment investigating the expression profiles of long non-coding RNAs (lncRNAs) in mice with high-altitude hypoxia-induced brain injury using a microarray technique revealed that *Gymnadenia conopsea* may regulate lncRNAs by acting on targets such as HMGA2, SRY, GATA4, SOX5, and ZBTB16, and modulating the HIF-1, PI3K-Akt, and NF-kappa B signaling pathways to achieve anti-inflammatory effects and adapt to hypoxia (Zhang et al., 2020). The above-described evidence suggests that *Gymnadenia conopsea* may provide a new research direction for interventions to treat HACE, but its specific molecular mechanisms needs to be further verified in high-altitude hypoxia animal models.

*Gynostemma pentaphyllum* is a herbaceous climbing plant of the Cucurbitaceae family. As a plant outside the Araliaceae family that contains saponins, which are similar in structure to ginsenosides, its saponin components have pharmacological effects, including protecting the cardiovascular and cerebrovascular systems, and anti-cerebral hypoxia, antitumor, and anti-aging effects, with therapeutic value in the clinical treatment of hyperlipidemia, vascular dementia, peptic ulcer, and stroke. Findings from an *in vivo* animal-based experiment suggest that the biologically active compound gypenoside (GP-14) mitigates neuroinflammation and BBB disruption in a mouse model of HACE. It achieves this by suppressing the NF-κB signaling pathway (Geng Y. et al, 2022). Furthermore, GP-14, which functions as a neuroprotective agent, could avert neuronal damage caused by severe hypoxia. Given its properties, it shows great promise as a compound for the development of neuroprotective medications (Geng Y.-N. et al, 2022).

### 3.3 Chinese medicinal compound formulas

Shengmai San (SMS), a traditional Chinese herbal formulation comprising three key ingredients: *Panax ginseng*, *Ophiopogon japonicus*, and *Schisandra chinensis,* is traditionally used for regulating cardiopulmonary function and anti-fatigue treatment. Pharmacological studies have reported that this compound can significantly enhance the activity of SOD and reduce malondialdehyde (MDA) levels, to effectively alleviate oxidative stress damage and protect the BBB while reducing indicators of inflammation (Li L. H. et al, 2013). Mechanistic studies have reported that ginsenosides, as the core active components of SMS, have neurotrophic and neuroprotective effects. They exert antioxidant activity and regulate cell apoptosis through signaling pathways such as Akt and Nrf2/HO-1. Moreover, they modulate the cell cycle, proliferation, differentiation, and regeneration of nerve cells by regulating pathways such as NF-κB and MAPK. They downregulate the release of NF-κB-mediated inflammatory factors (TNF-α, IL-6), reduce the abnormal expression of neuronal apoptosis-related proteins, and maintain the integrity of the BBB, thereby exerting neuroprotective effects (Xie et al., 2018). Experimental data suggest that this formula provides a strategy for the prevention and treatment of HACE by intervening in the oxidative-inflammatory cascade and energy metabolism disorders through multi-target mechanisms.

Xuefu Zhuyu Decoction (XFZYD), a classic blood-activating and stasis-eliminating compound composed of persicae semen, carthami flos, chuanxiong rhizoma, paeoniae rubra-, angelicae sinensis-, and bupleuri radix, and others, is used clinically to improve diseases related to microcirculation disorders. Animal experiments have confirmed that XFZYD can improve neurological function in rats, reduce brain edema, and improve the morphology of surrounding cells; these mechanisms may be related to platelet activation and the PI3K-Akt signaling pathway (Zhou et al., 2020). XFZYD can significantly improve spatial memory in rats with traumatic brain injury and reduce the modified neurologic severity score and arachidonic acid, TNF-α, and IL-1β levels. After the application of XFZYD, a significant downregulation of AKT/mTOR/p70S6K proteins was observed in brain tissue, indicating that XFZYD can provide neuroprotection by exerting anti-inflammatory effects through the inhibition of the PI3K-AKT-mTOR pathway (Xing et al., 2016). Furthermore, XFZYD improves BBB damage by reducing the levels of MMP-9 and COX-2. Further exploration through metabolomics has shown that XFZYD may protect the BBB by regulating adenosine metabolism through equilibrative nucleoside transporter 2 (Wang et al., 2025). The above evidence suggests that XFZYD can protect neurons through anti-inflammatory, microcirculation-improving, and BBB-protecting effects, and constitutes a multi-dimensional intervention strategy for the prevention and treatment of HACE.

Angong Niuhuang Wan (AGNHW), an emergency compound composed of bufonis venenum, moschus, margarita, coptidis rhizoma, gardeniae fructus, and others, is traditionally used for clinical intervention in febrile diseases with delirium, stroke, and CNS injuries. Experiments have confirmed that AGNHW has a significant alleviating effect on LPS-induced cerebral vascular edema, and its mechanism is related to the inhibition of VE-cadherin degradation, blockade of caveolin-1 phosphorylation, and regulation of the expression of AQP4 in astrocyte membranes (Liu et al., 2024). AGNHW can protect BBB integrity and improve neurological function by inhibiting the activation of MMP-9, mediated by peroxynitrite (Chen et al., 2022). Additionally, the active components in the compound can exert neuroprotective effects by inhibiting LPS-induced dopamine uptake reduction, reducing intracellular ROS production, and inhibiting the release of inflammatory factors such as TNF-α and IL-6 (Zhang F. et al, 2010). The above evidence indicates that AGNHW alleviates cerebral edema and exerts neuroprotective effects by synergistically regulating vascular permeability, BBB integrity, and inflammatory responses, providing a multidimensional pharmacological basis for the prevention and treatment of HACE.

Tianma Gouteng Yin (TMGTY), a classic Chinese medicinal compound composed of gastrodia elata, uncariae ramulus cum uncis, concha ostreae, gardeniae fructus, scutellariae radix, and others, has the core clinical efficacy of calming the liver and extinguishing wind, clearing heat, and reducing blood pressure. The aqueous extracts of the main components, gastrodia and uncaria, in TMGTY can upregulate Bcl-2 expression, reduce caspase-3 activity, and demonstrates antioxidant and anti-apoptotic capabilities *in vitro* and *in vivo*, thereby reducing the infarct area in rats with MCAO, maintaining tissue integrity, and exerting neuroprotective effects (Xian et al., 2016). Uncaria contains active indole and oxindole alkaloids that can cross the BBB and have antioxidant, anti-inflammatory, and neuroregulatory activities, with therapeutic effects on cardiovascular and CNS diseases (Kushida et al., 2021). These studies have reported that TMGTY has anti-inflammatory, antioxidant, anti-apoptotic, and neuroprotective effects, providing a potential pharmacological basis for its use in HACE.

## 4 Progress in research on treating HACE with TM

### 4.1 Understanding of HACE in TM

TM is a traditional healthcare system based on the theory of the “dynamic balance of three humors,” which advocates that the coordinated functioning of three physiological systems—energy circulation (wind), metabolic regulation (bile), and structural stability (phlegm)—maintain human health. This medical system, born on the Tibetan Plateau, relies on the unique medicinal resources of the cold regions (over 80% of which are plateau plants), integrating ecology with clinical practice to form a unique diagnostic and therapeutic model (Fu et al., 2020; Wang and Zhu, 2024). Its core concept emphasizes the high correlation between human physiology and high-altitude environments, providing an important basis for studying adaptability medicine at high altitudes (Schwabl and Vennos, 2015).

In TM, HACE is considered a systemic imbalance triggered by the cold and hypoxic environment. The Tibetan medical classic “Four Medical Tantras” indicates that the cold, hypoxia, and sudden changes in atmospheric pressure in high-altitude areas can easily lead to excessive activity of the energy circulation system (wind), thereby disturbing blood circulation and neuroregulatory functions (Yu et al., 2026). This pathological state manifests as brain hypoxia, fluid metabolic disorders, and abnormal neural signal transmission, which highly corresponds to the cerebral tissue edema and microcirculation disorders described in modern medicine. TM observes that such imbalances are frequently accompanied by symptoms such as severe headache, confusion, and vomiting, and are essentially the result of “uncontrolled energy circulation” and “obstructed qi and blood pathways.” TM treatment is based on the principles of “restoring the balance of the three humors” and “unblocking physiological pathways,” which focuses on multi-target intervention. For example, compound preparations, such as Duoxuekang capsules and Qishiwei Zhenzhu Pills, improve cerebral microcirculation, regulate neuro-metabolism, and enhance oxygen-utilization efficiency through synergistic effects. Modern research has confirmed that the active components in these drugs (e.g., salidroside and crocin) have anti-hypoxia, anti-inflammatory, and neuroprotective effects, and their compound combinations can significantly alleviate brain edema and reduce the risk of recurrence (Luo et al., 2022a). This holistic treatment strategy based on environmental adaptability reflects the diagnostic wisdom of the “cause–environment–constitution” TM triad and provides innovative ideas for the prevention and treatment of high-altitude cerebral diseases.

### 4.2 Individual Tibetan botanical drugs

*Potentilla anserina L.*, a characteristic Tibetan medicinal resource of the Tibetan Plateau, has unique advantages in the medical intervention of HACE with its active metabolites; potentilla anserina polysaccharide (PAP), euscaphic acid, and tormentic acid. PAP can significantly reduce brain tissue water content and improve neuronal damage through multi-target regulation, mainly via dual inhibition of the NF-κB and HIF-1α signaling pathways. PAP blocks the NF-κB signaling pathway to downregulate the expression of pro-inflammatory cytokines such as TNF-α and IL-6, inhibiting the neuroinflammatory cascade, while also inhibiting the hypoxia stress signals mediated by HIF-1α, reducing abnormal secretion of VEGF, thereby decreasing vascular permeability and the risk of brain edema (Shi J. et al, 2020). Euscaphic acid can protect vascular endothelial cells from hypoxia-induced apoptosis through the ERK1/2 signaling pathway, and tormentic acid can fully exert its therapeutic effects through the PI3K/AKT and ERK1/2 signaling pathways (Shi C. et al, 2020). Thus, *P. anserina* and its active metabolites provide a pharmacological basis for the prevention and treatment of HACE that is characterized by regional adaptability and multi-target regulation.

*Rhodiola crenulata* is a representative botanical drug in the TM system and is also included in the TCM system. This plant has demonstrated significant neuroprotective effects in preventing and treating brain damage caused by high-altitude hypoxia, reflecting its unique value in the cross-application of ethnic medicines. *Rhodiola crenulata* improves brain damage induced by HH through a multi-target regulatory mechanism. Its extracts and core metabolite salidroside can inhibit the NF-κB/NLRP3 signaling pathway, downregulate the expression of proinflammatory factors, and reduce neuroinflammation. Moreover, they can alleviate pathological damage and oxidative stress responses that are induced by HH, and this is mediated by reducing ROS and MDA levels and increasing SOD and GSH-Px activities (Xie et al., 2022). Salidroside can partially enhance energy metabolism while reducing the release of LDH and LD and reversing the degradation of the tight junction proteins ZO-1, occludin, and claudin-5. These data indicate that *R. crenulata* and its active metabolites can inhibit the NF-κB/NLRP3 pathway to reduce HH-induced brain oxidative stress damage, inflammatory responses, and BBB damage, which are attributed to improved energy metabolism and the anti-inflammatory M2 microglial phenotype (Jiang et al., 2022). *Rhodiola crenulata* and salidroside can exert anti-hypoxia neuroprotective effects by inhibiting neuronal apoptosis, maintaining BBB integrity, increasing tight junction proteins claudin-1, ZO-1, and occludin, and improving mitochondrial morphology and function, possibly through activation of the AMPK/Sirt1 signaling pathway (Hou et al., 2024). *Rhodiola crenulata* extracts exert antioxidant effects by scavenging lipid peroxidation products (e.g., MDA) and enhancing SOD activity, regulating cell apoptosis and mitochondrial energy metabolism through the HIF-1α/microRNA 210/ISCU1/2 (COX10) signaling pathway, and inhibiting caspase-3 activation to improve brain tissue energy metabolism disorders to alleviate HH-induced brain damage (Wang et al., 2019).

*Crocus sativus* is an important component of traditional TM, and its active metabolites crocin demonstrates significant neuroprotective effects in the treatment of HACE. Crocin, a glycoside of the natural crocin found in *Crocus sativus*, has antioxidant, anti-inflammatory, and antibacterial activities. Crocin can inhibit the expression of tight junction proteins, enhance the loss of nicotinamide adenine dinucleotide phosphate oxidase, block the induction of MMP-2 and MMP-9 by cerebral ischemia, thereby exerting antioxidant and BBB integrity-maintaining effects (Zhang et al., 2017). Crocin can reduce oxidative stress and inhibit inflammatory responses to protect brain tissue in rats with high-altitude hypoxia (Yang et al., 2024). Animal experiments have confirmed that crocin treatment can reduce inflammation and brain damage after hypoxia-ischemia and exerts neuroprotective effects (Huang and Jia, 2021).

*Saussurea involucrata* is a perennial herb of the Asteraceae family that is mainly distributed in high-altitude areas of western China. As a plant growing in high-altitude regions, it is endowed with distinctive physiological mechanisms and stress-responsive genes that enable it to adapt to high-altitude environments. Transcriptomic and metabolomic analyses of *S. involucrata* leaves revealed that *S. involucrata* responds to HH by participating in various metabolic activities related to deoxyribonucleic acid repair, membrane transport, hypoxia response, reproductive processes, and the effective utilization of nutrients and metabolites. These discoveries lay a theoretical groundwork for harnessing its medicinal potential and facilitating its cultivation in low-altitude regions (Quan et al., 2025). *Saussurea involucrata* cold-resistant characteristics adapt to extreme climates, and the whole herb contains active metabolites with antitumor, anti-inflammatory, analgesic, antioxidant, anti-fatigue, anti-aging, anti-hypoxia, neuroprotective, and immunomodulatory effects, making it an important resource for traditional TM and modern drug development (Chik et al., 2015). The petroleum ether extract of *S. involucrate*, when administered to mice, significantly reduced mortality under acute decompression conditions. Under chronic decompression conditions, changes in biochemical indicators of sugar metabolism and energy metabolism, such as adenosine triphosphate (ATP) content and ATPase activity in the brain and lactate (LAC) and lactate dehydrogenase (LDH) in the blood, were reversed. It exhibits high anti-hypoxia activity and can effectively prevent HACE (Ma et al., 2011).

*Brassica rapa L.*, a traditional TM derived from the Brassicaceae family, has been widely used to treat high-altitude diseases. As listed in the famous century-old Tibetan medical classic “Four Medical Tantras,” its roots and leaves are commonly used as medicines with anti-hypoxia, anti-fatigue, analgesic, anti-inflammatory, and neuroprotective effects. Polysaccharides from *Brassica rapa L.* are important metabolites in TM, and they have a significant protective effect on brain damage induced by acute high-altitude hypoxia. The mechanism may be related to the improvement of oxidative stress damage, inhibition of cell apoptosis through the activation of the PI3K/Akt/HIF-1α signaling pathway, and reversal of metabolic pathway disorders (Zou et al., 2022). The aqueous extract of *B. rapa* can increase the survival rate of HT-22 cells damaged by OGD/R by activating the PI3K/Akt/mTOR pathway, reduce cerebral infarct volume, and exert neuroprotective effects (Hua et al., 2021).

### 4.3 Prescriptions of TM

Duoxuekang (DXK) capsules are an empirical prescription in TM for treating high-altitude brain damage induced by HH. DXK can lower hematological indices, inhibit erythropoietin, affect the MAPK signaling pathway in oxidative damage, and regulate the RAS signaling pathway to exert brain-protective effects (Chen et al., 2021). DXK exhibits neuroprotective effects against HH-induced brain injury that is mediated through multiple mechanisms, such as enhancing cerebral blood perfusion, boosting the quantity of collagen and elastic fibers in brain tissues, and curbing oxidative stress-related damage. The underlying mechanism might be associated with safeguarding the integrity of cerebral vascular endothelial cells and preserving normal vascular function (Li et al., 2021).

Qishiwei Zhenzhu Pills (QSW) is a well-known traditional Tibetan medicinal formulation whose earliest documentation can be traced back to the revered Tibetan medical masterpiece, *The Four Medical Tantras*. For centuries, QSW has been employed in the therapeutic management of various ailments, including stroke, paralytic conditions, hemiplegia, and cerebral hemorrhage (Fu et al., 2022). QSW can dredge meridians, harmonize qi and blood, restore consciousness, induce resuscitation, improve cerebral blood vessels, protect neurons, and enhance learning and memory (Yan et al., 2022). In a cerebral I/R injury rat model, after pretreatment with QSW, neurological function was improved, infarct volume was reduced, Nissl bodies increased, tissue pathology was improved, and BBB disruption was decreased (Xu et al., 2020). QSW can lessen the intensity of cerebral I/R-induced injury by modulating the composition of the gut microbiota and suppressing inflammatory reactions in the body (Fu et al., 2021). Therefore, QSW is a good medicine for improving brain damage, and can play a certain role in the prevention and treatment of high-altitude cerebral diseases.

Ershiwuwei Shanhu Pills (ESP) contain components such as coral, crocus, and cinnamon, and is a classic TM formula that is mainly used to treat nervous system diseases, especially neurological pain and epilepsy. ESP can also improve microcirculation disorders in HACE (Ruan et al., 2022). Coral, the main component of ESP, has anticancer, anti-inflammatory, analgesic, antibacterial, antiviral, immunosuppressive, antioxidant, and neurological properties, and is widely used to treat nervous system diseases such as migraine, primary headache, epilepsy, cerebral infarction, hypertension, and other cardiovascular and cerebrovascular diseases (Han et al., 2023). ESP exhibits a protective effect on mice via the Keap1/Nrf2/HO-1 signaling cascade, shielding them from learning and memory impairments triggered by scopolamine (Zhang B.-Y. et al, 2022). In a mouse model of Alzheimer’s disease established by exposure to a combination of D-galactose and aluminum chloride, administration of ESP mitigated the deterioration of learning and memory capacities, and oxidative stress-induced damage, possibly via modulation of the Akt/mTOR/GSK-3β signaling pathway (Luo et al., 2022b).

## 5 Limitations of research and future directions

Further comparative analysis revealed that TCM and TM share several similarities and exhibit distinct differences in the treatment of HACE. Both systems emphasize holistic regulation of the body’s state, and some of their core mechanisms of action are highly overlapping. For instance, TCM herbs such as Coptis chinensis, Curcuma longa, Gynostemma pentaphyllum, and SMS, as well as Tibetan medicinal materials like Potentilla anserina, Rhodiola crenulata and its derivatives, all exert neuroprotective effects and maintain the integrity of the BBB by regulating signaling pathways such as NF-κB, inhibiting the release of inflammatory cytokines and alleviating oxidative damage. In terms of differences, TCM prioritizes rapid symptom relief by regulating the circulation of qi and blood. In contrast, TM focuses more on utilizing native high-altitude medicinal materials, such as Rhodiola rosea, to directly enhance the body’s hypoxia tolerance. Regarding synergy with modern conventional therapies, both TCM and TM can serve as important adjunctive treatments. When used in combination with Western medical interventions such as dehydration therapy and oxygen therapy, they can further reduce intracranial pressure and shorten the recovery time of neurological function. Moreover, TCM and TM can complement each other’s advantages, blocking the pathological process of HACE through multiple pathways and providing a more comprehensive approach for clinical treatment.

The exploration of TCM and TM in the treatment of HACE holds considerable promise; however, a major limitation is the lack of rigorous and high-quality scientific evidence for the treatment of HACE with TCM and TM. Reviews have reported valuable information on TCM for AMS; however, the focus is relatively broad, and specific evidence for the treatment of HACE with TCM remains limited. Currently, well-designed randomized controlled clinical trials for specific TCM formulas, TMs, acupuncture protocols, and other traditional HACE therapies, which employ rigorous Western medical diagnostic criteria and outcome measurement methods are lacking.

The current research on HACE mainly includes basic models (*in silico*, *in vitro*, *in vivo*), which have limitations in predicting the complex pathophysiology of the human body. Future mechanistic studies should include more models with greater translational significance, while addressing key issues such as pharmacokinetics.

In addition, methodological challenges to studying traditional medical systems such as TCM and TM exist. Standardization of herbal formulas which usually contain multiple components can be complex. The design and implementation of placebo controls for herbal interventions are also challenging. Moreover, TCM and TM emphasize individualized treatment based on traditional diagnostic patterns which may be difficult to translate into the standardized protocols commonly used in Western clinical trials. Integrating traditional clinical research diagnostic methods with Western diagnostic criteria and outcome indicators requires further research in precision medicine.

Future research directions could prioritize the combination of TCM theory with modern scientific methods such as network pharmacology and bioinformatics for screening drug targets and disease targets, to conduct carefully designed multi-center RCTs to evaluate the efficacy and safety of specific TCM and TM interventions for HACE, including herbal formulas, acupuncture, moxibustion, and combined methods. These trials should follow international standards for clinical research, using appropriate controls, feasible blinding procedures, standardized outcome measurements, and relevant TCM and TM diagnostic assessments. Mechanistic studies could be expanded to elucidate the pharmacological mechanisms of TCM and TM formulas and medicines in the context of HACE. This should include *in vitro* and *in vivo* studies, and potential human *ex vivo* trials aimed at exploring their specific effects on inflammation, oxidative stress, BBB integrity, neuronal apoptosis, cerebral blood flow, and cell metabolism. In the development and implementation of research and clinical practice, TCM and TM products should be strictly standardized and subjected to quality control to ensure batch consistency and product safety. Finally, collaboration between Western medical researchers, TCM practitioners, and TM doctors could be promoted to facilitate knowledge exchange, methodological development, and culturally sensitive research methods.

Although the academic value and dissemination influence of Chinese academic literature have shown a steady upward trend in recent years, room for improvement in its promotion and exposure on the international academic stage, as well as in the research quality of some of the literature exists. We conducted rigorous design and implementation in the literature screening stage. To minimize the potential systematic bias caused by the selection of a single database, we systematically included multiple mainstream academic databases in both Chinese and English, and we comprehensively retrieved Chinese and English literature related to the research topic. However, this study still has certain limitations. Future research can further expand the scope of literature inclusion to further enhance the comprehensiveness and representativeness of the study. Translating key TCM and TM literature and research findings into English and other international languages is crucial for broader dissemination and collaboration.

Addressing these limitations and pursuing future research directions is essential for rigorously assessing the potential of TCM and TM in HACE management and transforming traditional wisdom into evidence-based, clinically effective, and accessible therapeutic approaches.

In conclusion, HACE remains a significant clinical challenge, especially with the increasing global travel and activities at high altitudes. Although Western medicine provides valuable treatment methods such as oxygen therapy and pharmacological interventions to reduce intracranial pressure, limitations still exist particularly in resource-limited environments and in cases of severe, rapidly progressing disease. TCM and TM, with their long history of managing high-altitude related diseases and holistic treatment methods, offer promising complementary and alternative strategies for the prevention and treatment of HACE.

Research to date, particularly in AMS and related hypoxic conditions, indicates that TCM and TM interventions, mainly through herbal medicines, may exert beneficial effects on HACE by regulating inflammatory responses, reducing oxidative stress, improving cerebral blood flow and microcirculation, providing neuroprotection, and potentially affecting cell metabolism (Bailey et al., 2009; Wang et al., 2023). These mechanisms are consistent with the pathophysiological pathways identified in Western medical research, and provides a rational scientific basis for their therapeutic potential.

Nonetheless, the evidence base for TCM and TM in HACE is still significantly limited. An urgent need for rigorous, well-designed clinical trials and mechanistic studies to validate traditional claims, elucidate mechanisms of action, standardize treatments, and integrate these valuable traditional medical systems into evidence-based HACE management exists. Future research should prioritize collaborative, methodologically robust investigations to bridge Eastern and Western medical perspectives, while aiming to improve prevention, treatment, and long-term outcomes for individuals at risk of or affected by HACE worldwide. The combination of traditional wisdom with the rigor of modern science is key to fully realizing the therapeutic potential in the challenging clinical field of TCM and TM.
